# Tofogliflozin does not delay progression of carotid atherosclerosis in patients with type 2 diabetes: a prospective, randomized, open-label, parallel-group comparative study

**DOI:** 10.1186/s12933-020-01079-4

**Published:** 2020-07-09

**Authors:** Naoto Katakami, Tomoya Mita, Hidenori Yoshii, Toshihiko Shiraiwa, Tetsuyuki Yasuda, Yosuke Okada, Keiichi Torimoto, Yutaka Umayahara, Hideaki Kaneto, Takeshi Osonoi, Tsunehiko Yamamoto, Nobuichi Kuribayashi, Kazuhisa Maeda, Hiroki Yokoyama, Keisuke Kosugi, Kentaro Ohtoshi, Isao Hayashi, Satoru Sumitani, Mamiko Tsugawa, Kayoko Ryomoto, Hideki Taki, Tadashi Nakamura, Satoshi Kawashima, Yasunori Sato, Hirotaka Watada, Iichiro Shimomura, the UTOPIA study investigators

**Affiliations:** 1 Department of Metabolic Medicine, Osaka University Graduate School of Medicine, 2-2 Yamadaoka, Suita, Osaka 565-0871 Japan; 2 Department of Metabolism and Atherosclerosis, Osaka University Graduate School of Medicine, 2-2 Yamadaoka, Suita, Osaka 565-0871 Japan; 3 Department of Metabolism & Endocrinology, Juntendo University Graduate School of Medicine, Hongo 2-1-1, Bunkyo-ku, Tokyo, 113-8421 Japan; 4 Department of Medicine, Diabetology & Endocrinology, Juntendo Tokyo Koto Geriatric Medical Center, Koto-ku, Tokyo, 136-0075 Japan; 5 Shiraiwa Medical Clinic, 4-10-24 Hozenji, Kashiwara City, Osaka 582-0005 Japan; 6 Department of Diabetes and Endocrinology, Osaka Police Hospital, 10-31, Kitayama-cho, Tennoji-ku, Osaka, 543-0035 Japan; 7 First Department of Internal Medicine, School of Medicine, University of Occupational and Environmental Health, Japan, 1-1, Iseigaoka, Yahatanishi-ku, Kitakyushu, 807-8555 Japan; 8 Department of Diabetes and Endocrinology, Osaka General Medical Center, 3-1-56, Bandai-Higashi, Sumiyoshi-ku, Osaka, 558-8558 Japan; 9 Department of Diabetes, Endocrinology and Metabolism, Kawasaki Medical School, 577 Matsushima, Kurashiki, Okayama 701-0192 Japan; 10 Nakakinen Clinic, 745-5, Nakadai, Naka City, Ibaraki 311-0113 Japan; 11 Diabetes and Endocrinology, Kansai Rosai Hospital, 3-1-69, Inabaso, Amagasaki City, Hyogo Japan; 12 Misaki Naika Clinic, 6-44-9, Futawa-higashi, Funabashi City, Chiba Japan; 13 Kitasenri Maeda Clinic, 4-119 Furuedai, Suita, Osaka 565-0874 Japan; 14 Jiyugaoka Medical Clinic, West 6, South 6-4-3, Obihiro, Hokkaido 080-0016 Japan; 15 Kosugi Medical Clinic, 3-9, Tamatsukurimoto-cho, Tennoji-ku, Osaka, 543-0014 Japan; 16 Otoshi Medical Clinic, 8-47, Kakudacho, Osaka Kita-ku, Osaka, 530-0017 Japan; 17 Hayashi Clinic, 3-9-23 Koshienguchi, Nishinomiya, Hyogo 663-8113 Japan; 18 Center for Diabetes and Endocrinology, Nippon Life Hospital, 2-1-54 Enokojima, Nishi-ku, Osaka, 550-0006 Japan; 19 Department of Endocrinology and Metabolism, Ikeda Municipal Hospital, 3-1-18, Jonan, Ikeda, Osaka 563-8510 Japan; 20 Center for Diabetes Mellitus, Osaka Rosai Hospital, 1179-3 Nagasone-cho, Kita-ku, Sakai, Osaka 591-8025 Japan; 21 Diabetes Center, National Hospital Organization Osaka National Hospital, 2-1-14, Hoenzaka, Chuo-ku, Osaka, 540-0006 Japan; 22 Department of Internal Medicine, Kawasaki Hospital, 3-3-1, Higashiyamacho, Kobe Hyogo-ku, Hyogo, 652-0042 Japan; 23 Kanda Naika Clinic, 5-21-3, Hannancho, Osaka Abeno-ku, Osaka, 545-0021 Japan; 24 Department of Preventive Medicine and Public Health, Keio University School of Medicine, 45 Shinanomachi Shinjuku-ku, Tokyo, 160-8582 Japan

**Keywords:** Atherosclerosis, Diabetes, Intima-media thickness, SGLT2 inhibitor, Tofogliflozin

## Abstract

**Background:**

This study aimed to investigate the preventive effects of tofogliflozin, a selective sodium-glucose cotransporter 2 (SGLT2) inhibitor, on atherosclerosis progression in type 2 diabetes (T2DM) patients without apparent cardiovascular disease (CVD) by monitoring carotid intima-media thickness (IMT).

**Methods:**

This prospective, randomized, open-label, blinded-endpoint, multicenter, parallel-group, comparative study included 340 subjects with T2DM and no history of apparent CVD recruited at 24 clinical units. Subjects were randomly allocated to either the tofogliflozin treatment group (n = 169) or conventional treatment group using drugs other than SGLT2 inhibitors (n = 171). Primary outcomes were changes in mean and maximum common carotid IMT measured by echography during a 104-week treatment period.

**Results:**

In a mixed-effects model for repeated measures, the mean IMT of the common carotid artery (mean-IMT-CCA), along with the right and left maximum IMT of the CCA (max-IMT-CCA), significantly declined in both the tofogliflozin (− 0.132 mm, SE 0.007; − 0.163 mm, SE 0.013; − 0.170 mm, SE 0.020, respectively) and the control group (− 0.140 mm, SE 0.006; − 0.190 mm, SE 0.012; − 0.190 mm, SE 0.020, respectively). Furthermore, the tofogliflozin and the conventional treatment group did not significantly differ in the progression of the mean-IMT-CCA (mean change (95% CI) 0.008 (− 0.009, 0.025) mm, P = 0.34), along with the right (mean change (95% CI) 0.027 (− 0.005, 0.059) mm, P = 0.10) and the left max-IMT-CCA (mean change (95% CI) 0.020 (− 0.030, 0.070), P = 0.43). Similar findings were obtained even after adjusting for traditional CV risk factors and/or administration of drugs at baseline. Relative to the control treatment effects, tofogliflozin significantly reduced the HbA1c, blood glucose level, body weight/body mass index, abdominal circumference, and systolic blood pressure, and significantly increased the HDL-C. The total and serious adverse events incidences did not significantly vary between the treatment groups.

**Conclusions/interpretation:**

No IMT changes were observed between the tofogliflozin and the conventional treatment groups. However, tofogliflozin is a safe and effective treatment option for managing primary CVD risk factors in this population.

*Clinical Trial Registration* UMIN000017607 (https://www.umin.ac.jp/icdr/index.html).

## Background

Sodium-glucose cotransporter 2 (SGLT2) inhibitors are antidiabetic agents that lower blood glucose levels by promoting urinary glucose excretion. Their risk of causing hypoglycemia, which is linked to increased cardiovascular (CV) events [1–3], is low since their mode of action is independent of insulin secretion. SGLT2 inhibitors are known to diminish various CV risk factors by reducing visceral adipose tissue, body weight, and blood pressure, improving the blood lipid profile, and generating a reno-protective effect independent of the glycemic effects [4, 5]. Because SGLT2 inhibitors have a pleiotropic antiatherogenic effect, they are expected to attenuate the progression of atherosclerosis, and therefore, to protect against CV events.

Clinical trials in patients with type 2 diabetes mellitus (T2DM) showed that SGLT2 inhibitors, such as empagliflozin and canagliflozin, significantly reduced the primary outcome, a composite of death from CV causes, nonfatal myocardial infarction, and nonfatal stroke, compared to that of placebo [6, 7]. However, although worsening heart failure was decreased, these treatments failed to reduce atherothrombotic events, such as myocardial infarction and stroke [5–9]. Thus, clinical evidence of the anti-atherosclerotic effect of SGLT2 inhibitors remains to be established. Furthermore, to our knowledge, only very few clinical trials have investigated whether SGLT2 inhibitors protect against atherosclerosis in subjects with T2DM but no apparent cardiovascular disease (CVD) [10].

Tofogliflozin, an SGLT2 inhibitor that has been clinically used in Japan since 2014, is associated with favorable metabolic effects, including improved glycemic control and serum lipid profile, along with decreased body weight, visceral adipose tissue, and blood pressure [11, 12]. Tofogliflozin has the highest selectivity of all clinically developed inhibitors with 2900-fold greater selectivity for SGLT2 than SGLT1 [13], which may contribute to the relatively low incidence of adverse events including hypoglycemia, compared to that of other SGLT2 inhibitors [14]. Moreover, among all SGLT2 inhibitors, tofogliflozin has the shortest half-life with a urinary excretion rate of more than 80% within 12 h after administration. Morning tofogliflozin administration reportedly reduces the risk of nocturnal hypoglycemia because its effects almost disappear by nighttime [15].

In patients with T2DM, a progressive thickening of the carotid artery intima-media is considered a CVD surrogate marker [16] used for evaluating the effects of various interventions on the progression of atherosclerosis [17–24]. Our randomized controlled trial investigated the preventive effects of tofogliflozin on the progression of intima-media thickness (IMT) in patients with apparent CVD-free T2DM.

## Methods

### Study design

The Study of Using Tofogliflozin for Possible better Intervention against Atherosclerosis for type 2 diabetes patients (UTOPIA) trial was a multicenter prospective, randomized (1:1), open-label, blinded-endpoint (PROBE) study, as described previously [25]. This study is registered in the University Hospital Medical Information Network Clinical Trials Registry (UMIN-CTR), a nonprofit organization in Japan, and meets the requirements of the International Committee of Medical Journal Editors (UMIN000017607).

### Study population

Japanese subjects with T2DM who periodically attended the outpatient diabetes clinics of 24 institutions in Japan (Additional file 1: Material S1) were asked to participate in this study, as described in detail previously [25]. The inclusion criteria were as follows: (1) Japanese with T2DM and inadequate glycemic control (HbA1c ≥ 6% but < 9%), along with the inability to achieve the blood glucose level stated in the Diabetes Treatment Guideline of 2014–2015 despite being on drugs—except SGLT2 inhibitors—with diet and physical therapy, on diet and physical therapy without being on drugs for at least 12 weeks, or on SGLT2 inhibitors in the past but without them for at least 12 weeks before signing the consent form, (2) without changes (including new prescriptions) in the antidiabetic, antithrombotic, antihypertensive medication, or a therapeutic agent for dyslipidemia management for at least 12 weeks before signing the consent form, (3) age 30–74 at the time of giving consent, and (4) able to provide informed consent. Furthermore, the following exclusion criteria were applied: (1) type 1 or secondary diabetes, (2) in the perioperative period or with a serious infection or injury, (3) a history of myocardial infarction, angina, stroke, or cerebral infarction, (4) severe renal dysfunction (estimated glomerular filtration rate (eGFR) of < 30 mL/min/1.73 m^2^) or end-stage renal failure (eGFR < 15 ml/min/1.73 m^2^, i.e., dialysis or renal transplantation is required), (5) serious liver functional impairment (aspartate aminotransferase ≥ 100 U/L), (6) moderate to severe heart failure (class 3 or worse based on the New York Heart Association Functional Classification), (7) urinary tract or genital infection, (8) pregnant, possibly pregnant, nursing, or planning to conceive a child, (9) history of hypersensitivity to the study drug, (10) present or past history of a malignant tumor (exceptions: patients not on medication for malignant tumor and no recurrence of the disease so far without recurrence risks during this study were allowed to participate), (11) prohibited to use tofogliflozin, (12) other ineligibility determined by an investigator.

The subjects were screened consecutively, and patients who met the above eligibility criteria were asked to participate in our study. All patients who agreed to participate were included in the study. The protocol was approved by the Osaka University Clinical Research Review Committee and the institutional review board of each participating institution in compliance with the Declaration of Helsinki and current legal regulations in Japan. Written informed consent was obtained from all participants after a full explanation of the study.

### Randomization and study intervention

Patient registration was performed at the administration office of the UTOPIA trial via the internet; once enrolled, the subjects were randomly and equally assigned to a tofogliflozin treatment group or a conventional treatment group using drugs other than SGLT2 inhibitors. The randomization was performed using a dynamic balancing minimization method based on insulin use/non-use, age, and sex. Assignments were made by the electronic data capturing system using computer-generated random numbers and minimization software for group allocation. The computer programs for analyses were developed and run by biostatisticians, following the prespecified statistical analysis plan. Neither patients nor investigators were masked to treatment group assignment.

Treatment was continued to achieve the target value specified in the Japanese treatment guide for diabetes [26] (generally an HbA1c < 7.0%) in all patients. In the conventional treatment group, either the current therapy dosage was increased or a concomitant oral glucose-lowering drug (excluding any other SGLT2 inhibitor) was added 12 weeks following randomization. In the tofogliflozin group, 20 mg tofogliflozin once daily was started in addition to ongoing therapy. However, the addition of an alternative antidiabetic agent (excluding another SGLT2 inhibitor) was permitted 12 weeks after randomization. In the case of hypoglycemia, the dosage of the concomitant oral glucose-lowering drug was titrated. The use of antihyperlipidemic and antihypertensive drugs was allowed during the study.

### Observation items and schedule

The study period was 104 weeks following patient registration (registration period: January to November 2016). All randomized participants were followed until the scheduled study end regardless of adherence to or discontinuation of study medication for any reason. Clinical outcomes, adherence, and adverse events were confirmed, and clinical and biochemical data were collected at 0, 26, 52, 78, and 104 weeks after randomization.

### Study outcomes

Primary study outcomes were the changes in mean IMT of the common carotid artery (mean-IMT-CCA) and maximum IMT of the CCA (max-IMT-CCA) during the 104-week treatment period measured by carotid arterial echography. The most primary outcome of this study was preliminarily defined as the change in the mean derived from the left- and right-side mean-IMT-CCA values. Investigations were conducted at the beginning of the study and at 52 and 104 weeks.

### Measurement of carotid IMT

Ultrasonography scans of the carotid artery based on the guideline of the Japan Society of Ultrasonics in Medicine [27] were performed by expert sonographers specifically trained to perform the prescribed study examination. To avoid inter-sonographer variability, each participant was examined by the same sonographer with the same equipment (high-resolution B-mode ultrasound scanner equipped with a high-frequency [> 7.5 MHz] linear transducer with a limit of detection of < 0.1 mm) throughout all the visits. Scanning of the extracranial CCA, the carotid sinus, and the internal carotid artery in the neck was performed bilaterally in at least three different longitudinal projections as well as transverse projections, and the site of greatest thickness, including plaque lesions, was sought along the arterial walls. The IMT was measured as the distance between two parallel echogenic lines corresponding to the vascular lumen and the adventitial layer.

To avoid inter-reader variability, all scans were electronically stored and sent to the central office (IMT Evaluation Committee, Osaka, Japan) for reading by a single experienced reader unaware of the subjects’ clinical characteristics in a random order using automated digital edge-detection software (Intimascope; MediaCross, Tokyo, Japan) [28]. The software system averaged about 200 points of IMT values in the segment 2 cm proximal to the dilation of the carotid sinus (mean-IMT-CCA). In addition, the maximum thicknesses of the intima and media layers, including the plaque lesions, in the common carotid arteries (max-IMT-CCA) were captured separately. The same systematic procedures for analyzing carotid IMT were used in our previous studies [21, 22]. Reproducibility analysis of replicate measurements in the randomly selected 20 subjects yielded absolute mean differences of 0.02 ± 0.01 and 0.01 ± 0.01 for mean-IMT-CCA and max-IMT-CCA, respectively. The intra-observer coefficients of variation for the measurements of mean-IMT-CCA and max-IMT-CCA were 1.1% and 0.7%, respectively.

### Biochemical tests

Blood samples were collected after overnight fasting. Serum lipids (total cholesterol, high-density lipoprotein-cholesterol, low-density lipoprotein-cholesterol, triglycerides), HbA1c (National Glycohemoglobin Standardization Program), glucose, insulin, and creatinine were measured with standard techniques. Measurements of highly sensitive C-reactive protein were outsourced to a private laboratory (SRL Laboratory, Tokyo). Urinary albumin excretion (UAE) was measured by the improved bromocresol purple method using a spot urine sample. The estimated glomerular filtration rate (eGFR) was calculated using the following formula: eGFR (ml/min per 1.73 m^2^) = 194 × age − 0.287 × serum creatinine − 0.1094 (× 0.739 for females) [29].

### Safety evaluation

All adverse events (AEs) were recorded during the study, as described in the Additional file 1.

## Sample size

The progression of carotid IMT in diabetic patients is considered to be 0.034 ± 0.054 mm/year (mean ± standard deviation [SD]), and a 1% improvement in the HbA1c value is associated with a 0.02 mm/year improvement in IMT [30]. Therefore, during a 2-year observation period, the registration of at least 310 patients was required to obtain 90% power to detect a difference of 0.04 mm in IMT between the two treatment groups assuming an SD of 0.108 mm for individual differences, which was presumed to be common in both groups, and a 0.05 level of significance. The dropout and/or study discontinuation rate during the 2-year observation period was assumed to be 10%. According to this calculation, the target number of enrolled patients was set at 340 (170 per group) for the 2-year registration period.

### Statistical analysis

All allocated participants, except those without any IMT measurements during the observation period, were analyzed regardless of adherence using an intent-to-treat approach. Analyses of the efficacy were performed on the full dataset using the intent-to-treat approach principle and secondarily using the per-protocol set. Primary analysis was performed using the mixed-effects model for repeated measures with treatment group, time (week), interactions between treatment group and time (week), age, sex, use of insulin at baseline, and baseline IMT as fixed effects; an unstructured covariate was used to model the covariance of within-subject variability. The sensitivity analysis assessed differences in delta change in IMT from baseline between two groups using analysis of covariance (ANCOVA) models that included treatment group, age, sex, baseline IMT, systolic blood pressure, and administration of statin. For the occurrence of cardiovascular events as one of the secondary outcomes, the time to onset was analyzed using a log-rank test and the Cox proportional hazard model.

Baseline and follow-up group comparisons were performed with Student’s *t* test or Wilcoxon rank-sum test for continuous variables and Fisher’s exact test or Chi square test for categorical variables. Changes from baseline to treatment visits were assessed with a one-sample t-test and Wilcoxon signed-rank test within the group. The frequency and proportion of patients reporting AEs were derived from each treatment group and compared between the two treatment groups using Fisher’s exact test. All statistical tests were two-sided with a 5% significance level. All analyses were performed using the SAS software version 9.4 (SAS Institute, Cary, NC).

### Role of the funding source

The sponsor had no role in study design, data collection, data analysis, data interpretation, or writing of the report. The corresponding author had full access to all the data in the study and had final responsibility for the decision to submit for publication.

## Results

### Study population

**Fig. 1:**
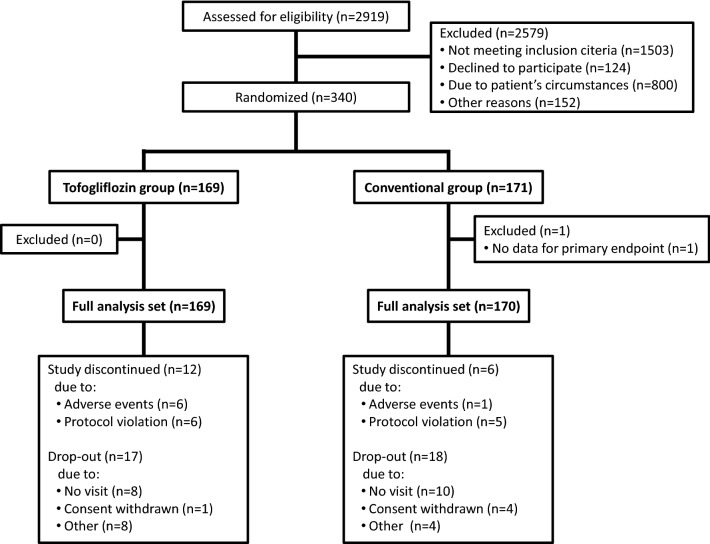
Study design flowchart

Between January 12, 2016, and November 25, 2016, 340 participants were randomly allocated to either the tofogliflozin group (n = 169) or the conventional treatment group (n = 171). After excluding 1 patient from further analysis because of no data for the primary endpoint, 169 and 170 patients of the tofogliflozin group and the conventional treatment group were included in the full analysis set, respectively. Among the study subjects, 140 of the tofogliflozin group and 146 of the conventional treatment group completed the allocated treatment regimen with the final patient visit on February 26, 2019 (Fig. 1). There were no significant differences in baseline characteristics between the two groups (Table 1).

**Table 1:** Clinical characteristics of patients in both treatment groups

Parameters	Tofogliflozin group	Conventional group	P value
Sex (males) (%)	99 (58.6)	99 (58.2)	1.00
Age (years)	61.3 ± 9.3	60.9 ± 9.7	0.66
Current smoking	38 (22.6)	29 (17.1)	0.22
Hypertension	88 (52.1)	105 (61.8)	0.08
Dyslipidemia	107 (63.3)	122 (71.8)	0.11
Duration of diabetes (years)	12.1 ± 8.4	12.5 ± 8.3	0.65
Diabetic retinopathy	28 (16.8)	33 (19.5)	0.51
Diabetic nephropathy	48 (28.4)	53 (31.2)	0.58
Use of glucose-lowering agents	153 (90.5)	152 (89.4)	0.86
Metformin	91 (53.8)	100 (58.8)	0.38
Sulfonylurea	38 (22.5)	43 (25.3)	0.61
Glinides	10 (5.9)	10 (5.9)	1.00
Thiazolidinediones	18 (10.7)	23 (13.5)	0.51
α-Glucosidase inhibitor	24 (14.2)	25 (14.7)	1.00
DPP-4 inhibitors	75 (44.4)	95 (55.9)	0.039
GLP-1 R agonists	23 (13.6)	12 (7.1)	0.05
Insulins	35 (20.7)	37 (21.8)	0.89
Use of antihypertensive drugs	79 (46.7)	95 (55.9)	0.10
Angiotensin-converting enzyme inhibitors	3 (1.8)	5 (2.9)	0.72
Angiotensin II receptor blockers	63 (37.3)	83 (48.8)	0.037
Direct renin inhibitor	2 (1.2)	0 (0.0)	0.25
Calcium channel blocker	47 (27.8)	54 (31.8)	0.48
Diuretic drugs	8 (4.7)	14 (8.2)	0.27
α-Adrenergic receptor antagonist	2 (1.2)	0 (0.0)	0.25
β-Adrenergic receptor antagonist	3 (1.8)	3 (1.8)	1.00
Others	5 (3.0)	10 (5.9)	0.29
Use of lipid-lowering agents	82 (48.5)	99 (58.2)	0.08
Statins	73 (43.2)	83 (48.8)	0.33
Ezetimibe	10 (5.9)	11 (6.5)	1.00
Resins	0 (0.0)	1 (0.6)	1.00
Fibrates	8 (4.7)	6 (3.5)	0.60
Use of antithrombotic agents	17 (10.1)	15 (8.8)	0.71
Antiplatelet agents	15 (8.9)	11 (6.5)	0.42
Anticoagulants	2 (1.2)	4 (2.4)	0.68
Others	0 (0.0)	0 (0.0)	–

Data are presented as number (%) of patients or mean ± SD values

### Carotid intima-media thickness

For 104 weeks, both tofogliflozin and conventional treatment significantly reduced the mean-IMT-CCA and the right and left max-IMT-CCA values relative to the respective baseline values (Table 2). In a mixed-effects model for repeated measures, there were no significant differences in the progression in the mean-IMT-CCA and the right and left max-IMT-CCA (i.e., primary endpoints of the study) between the tofogliflozin and the conventional treatment group (Table 2). Similar findings were obtained even after adjusting the mixed-effects models for traditional CV risk factors and/or administration of drugs, including hypoglycemic agents, antihypertensive agents, statin, and antiplatelets at baseline (Table 3). Moreover, ANCOVA models that included treatment group, age, sex, use of insulin, baseline IMT, systolic blood pressure, and administration of statins produced findings that resembled those generated by the mixed-effects models (Additional file 1: Table S1).

**Table 2:** Effects of tofogliflozin on intima-media thickness

	Tofogliflozin group	Conventional group	Treatment effect (tofogliflozin-conventional treatment) (mean change; 95%CI), P value	P value between groups
Common mean IMT				
Baseline (mm)	0.87 ± 0.16 (n = 169)	0.87 ± 0.16 (n = 170)		0.93
Week 52 (mm)	0.78 ± 0.14 (n = 155)	0.78 ± 0.14 (n = 160)		0.90
Week 104 (mm)	0.74 ± 0.14 (n = 152)	0.73 ± 0.14 (n = 149)		0.58
Week 52 (mean change; SE)	− 0.082 (0.006) §	− 0.083 (0.005) §	0.001 (− 0.012, 0.014), P = 0.89	
Week 104 (mean change; SE)	− 0.132 (0.007) §	− 0.140 (0.006) §	0.008 (− 0.009, 0.025), P = 0.34	
Right maximum IMT				
Baseline (mm)	1.05 ± 0.29 (n = 169)	1.06 ± 0.25 (n = 169)		0.76
Week 52 (mm)	0.96 ± 0.28 (n = 155)	0.95 ± 0.23 (n = 159)		0.75
Week 104 (mm)	0.91 ± 0.30 (n = 152)	0.88 ± 0.21 (n = 148)		0.41
Week 52 (mean change; SE)	− 0.107 (0.012) §	− 0.119 (0.011) §	0.013 (− 0.015, 0.040), P = 0.37	
Week 104 (mean change; SE)	− 0.163 (0.013) §	− 0.190 (0.012) §	0.027 (− 0.005, 0.059), P = 0.10	
Left maximum IMT				
Baseline (mm)	1.13 ± 0.37 (n = 169)	1.12 ± 0.37 (n = 169)		0.79
Week 52 (mm)	1.01 ± 0.34 (n = 155)	1.01 ± 0.33 (n = 159)		0.95
Week 104 (mm)	0.96 ± 0.38 (n = 152)	0.93 ± 0.31 (n = 148)		0.45
Week 52 (mean change; SE)	− 0.117 (0.018) §	− 0.108 (0.018) §	− 0.009 (− 0.053, 0.036), P = 0.70	
Week 104 (mean change; SE)	− 0.170 (0.020) §	− 0.190 (0.020) §	0.020 (− 0.030, 0.070), P = 0.43	

Data are presented as mean ± SD, unless stated otherwise. Comparisons of IMT values during treatment with those at baseline were performed using a one-sample t-test based on the mixed-effects model for repeated measures. *P < 0.05, ^#^P < 0.01, §P < 0.001. Differences in IMT between groups at each point were analyzed using Student’s t-test. Differences in delta change in IMT from baseline to week 52 and 104 between groups at each point (Treatment effect) were analyzed with the mixed-effects model for repeated measures. Treatment group, week, interactions between treatment group and week, age, sex, use of insulin at baseline, and baseline IMT were included as fixed effects. IMT, intima-media thickness

**Table 3:** Effects of tofogliflozin on intima-media thickness of common carotid arteries after adjusting for traditional CV risk factors and/or administration of drugs

	Model 1	Model 2	Model 3	Model 4
Common mean IMT				
Week 52	0.003 (− 0.013, 0.018)	0.001 (− 0.014, 0.017)	0.002 (− 0.014, 0.018)	0.002 (− 0.014, 0.017)
Week 104	0.008 (− 0.012, 0.028)	0.007 (− 0.013, 0.027)	0.008 (− 0.012, 0.028)	0.007 (− 0.013, 0.027)
Right maximum IMT				
Week 52	0.022 (− 0.010, 0.054)	0.019 (− 0.013, 0.051)	0.021 (− 0.011, 0.053)	0.021 (− 0.011, 0.053)
Week 104	0.030 (− 0.007, 0.066)	0.027 (− 0.010, 0.063)	0.028 (− 0.008, 0.065)	0.029 (− 0.007, 0.065)
Left maximum IMT				
Week 52	− 0.026 (− 0.077, 0.026)	− 0.029 (− 0.081, 0.023)	− 0.027 (− 0.079, 0.025)	− 0.028 (− 0.080, 0.024)
Week 104	− 0.006 (− 0.062, 0.050)	− 0.009 (− 0.065, 0.047)	− 0.007 (− 0.063, 0.049)	− 0.008 (− 0.064, 0.048)

Treatment effect (tofogliflozin—conventional treatment) is expressed as mean change (95% CI). Differences in delta change in IMT from baseline at 52 and 104 weeks between groups at each point (Treatment effect) were analyzed with mixed effects model for repeated measures. *P < 0.05, ^#^P < 0.01, §P < 0.001. Model 1: Treatment group, week, interactions between treatment groups and week, body mass index, HbA1c, total cholesterol, high-density lipoprotein-cholesterol and triglyceride and systolic blood pressure were included as fixed effects. Model 2: Model 1 plus smoking, DPP-4 inhibitors, pioglitazone, angiotensin-converting enzyme/angiotensin II receptor blocker, statin and anti-platelets were included as fixed effects. Model 3: Model 1 plus smoking, hypoglycemic agents, angiotensin-converting enzyme/angiotensin II receptor blocker, statin and anti-platelets were included as fixed effects. Model 4: Model 1 plus smoking, hypoglycemic agents, antihypertensive agents, statins, and antiplatelets were included as fixed effects

### Glucose metabolism and other parameters

Within 104 weeks, tofogliflozin treatment, but not conventional treatment, significantly reduced HbA1c and fasting blood glucose levels relative to baseline. The improvements (value at study end—value at baseline) in HbA1c (− 0.3 ± 0.8% vs. 0.1 ± 0.7%, P < 0.001) and fasting blood glucose (− 0.7 ± 1.9 mmol/l vs. 0.1 ± 1.8 mmol/l, P < 0.001) were significantly better in the tofogliflozin group than in the conventional group. The serum C-peptide level was also significantly decreased in the tofogliflozin group but not in the conventional group (Table 4).

**Table 4:** Effects of tofogliflozin on body mass index, glucose metabolism, lipid metabolism and blood pressure

Parameters	Tofogliflozin group	Conventional group	P value
Body Mass Index at baseline (kg/m^2^)	27.0 ± 5.8 (n = 168)	27.0 ± 4.6 (n = 169)	0.93
Week 26 (change from baseline)	− 0.7 ± 1.0 (n = 163)§	0.0 ± 1.2 (n = 164)	< 0.001
Week 52 (change from baseline)	− 0.8 ± 1.3 (n = 160)§	− 0.1 ± 1.4 (n = 159)	< 0.001
Week 78 (change from baseline)	− 0.8 ± 1.5 (n = 154)§	0.0 ± 1.5 (n = 157)	< 0.001
Week 104 (change from baseline)	− 1.0 ± 1.4 (n = 154)§	− 0.2 ± 1.8 (n = 154)	< 0.001
Waist circumference at baseline (cm)	93.0 ± 12.7 (n = 149)	93.7 ± 11.7 (n = 154)	0.60
Week 26 (change from baseline)	− 2.0 ± 6.0 (n = 128)§	1.0 ± 4.3 (n = 127)#	< 0.001
Week 52 (change from baseline)	− 0.9 ± 6.1 (n = 127)	1.4 ± 4.7 (n = 135)#	< 0.001
Week 78 (change from baseline)	− 1.3 ± 6.5 (n = 117)*	1.4 ± 4.3 (n = 124)§	< 0.001
Week 104 (change from baseline)	− 1.2 ± 6.0 (n = 124)*	1.5 ± 4.3 (n = 125)§	< 0.001
HbA1c at baseline (%)	7.4 ± 0.7 (n = 169)	7.3 ± 0.7 (n = 170)	0.23
HbA1c at baseline (mmol/mol)	57.5 ± 8.0 (n = 169)	56.5 ± 7.8 (n = 169)	0.23
Week 26 (change from baseline)	− 0.4 ± 0.6 (n = 165)§	0.0 ± 0.5 (n = 165)	< 0.001
Week 52 (change from baseline)	− 0.3 ± 0.7 (n = 161)§	0.0 ± 0.6 (n = 162)	< 0.001
Week 78 (change from baseline)	− 0.3 ± 0.8 (n = 154)§	0.0 ± 0.7 (n = 159)	< 0.001
Week 104 (change from baseline)	− 0.3 ± 0.8 (n = 156)§	0.1 ± 0.7 (n = 153)	< 0.001
Fasting blood glucose at baseline (mmol/l)	7.8 ± 1.7 (n = 167)	7.8 ± 1.8 (n = 168)	0.91
Week 26 (change from baseline)	− 0.9 ± 1.6 (n = 155)§	0.2 ± 2.1 (n = 152)	< 0.001
Week 52 (change from baseline)	− 0.8 ± 1.7 (n = 150)§	− 0.1 ± 1.8 (n = 154)	< 0.001
Week 78 (change from baseline)	− 0.5 ± 1.7 (n = 145)§	0.1 ± 1.9 (n = 146)	0.007
Week 104 (change from baseline)	− 0.7 ± 1.9 (n = 148)§	0.1 ± 1.8 (n = 149)	< 0.001
C-peptide at baseline (ng/ml)	1.91 ± 1.16 (n = 166)	2.04 ± 1.24 (n = 168)	0.34
Week 52 (change from baseline)	− 0.26 ± 0.78 (n = 149)§	− 0.20 ± 1.11 (n = 156)*	0.61
Week 104 (change from baseline)	− 0.16 ± 0.82 (n = 145)*	− 0.12 ± 1.06 (n = 149)	0.75
Total cholesterol at baseline (mmol/l)	4.96 ± 0.74 (n = 165)	4.93 ± 0.81 (n = 163)	0.72
Week 26 (change from baseline)	0.09 ± 0.55 (n = 156)*	− 0.03 ± 0.54 (n = 150)	0.07
Week 52 (change from baseline)	0.07 ± 0.59 (n = 157)	− 0.04 ± 0.57 (n = 156)	0.07
Week 78 (change from baseline)	0.09 ± 0.57 (n = 146)	− 0.05 ± 0.65 (n = 144)	0.05
Week 104 (change from baseline)	0.10 ± 0.69 (n = 151)	− 0.05 ± 0.67 (n = 147)	0.06
LDL cholesterol at baseline (mmol/l)	2.88 ± 0.69 (n = 168)	2.89 ± 0.66 (n = 169)	0.87
Week 26 (change from baseline)	0.03 ± 0.52 (n = 162)	− 0.02 ± 0.51 (n = 162)	0.47
Week 52 (change from baseline)	0.01 ± 0.50 (n = 159)	− 0.03 ± 0.51 (n = 162)	0.49
Week 78 (change from baseline)	0.04 ± 0.50 (n = 153)	− 0.05 ± 0.59 (n = 157)	0.14
Week 104 (change from baseline)	0.01 ± 0.62 (n = 154)	− 0.07 ± 0.57 (n = 153)	0.25
HDL cholesterol at baseline (mmol/l)	1.42 ± 0.36 (n = 169)	1.37 ± 0.31 (n = 170)	0.18
Week 26 (change from baseline)	0.07 ± 0.18 (n = 163)§	0.00 ± 0.18 (n = 162)	< 0.001
Week 52 (change from baseline)	0.06 ± 0.16 (n = 161)§	0.01 ± 0.18 (n = 162)	0.009
Week 78 (change from baseline)	0.08 ± 0.22 (n = 154)§	0.03 ± 0.20 (n = 159)	0.019
Week 104 (change from baseline)	0.08 ± 0.23 (n = 156)§	0.04 ± 0.20 (n = 154)*	0.08
Triglyceride at baseline (mmol/l)	1.20 [0.93, 1.80] (n = 167)	1.45 [1.01, 1.88] (n = 168)	0.06
Week 26 (change from baseline)	− 0.04 [− 0.34, 0.24] (n = 152)	0.03 [− 0.26, 0.36] (n = 150)	0.21
Week 52 (change from baseline)	− 0.03 [− 0.25, 0.23] (n = 149)	− 0.03 [− 0.37, 0.20] (n = 154)*	0.40
Week 78 (change from baseline)	− 0.01 [− 0.30, 0.23] (n = 145)	− 0.01 [− 0.34, 0.29] (n = 145)	0.82
Week 104 (change from baseline)	− 0.05 [− 0.36, 0.25] (n = 147)	− 0.03 [− 0.35, 0.30] (n = 148)	0.95
Systolic blood pressure (mmHg)	133.0 ± 14.5 (n = 165)	134.5 ± 17.4 (n = 165)	0.41
Week 26 (change from baseline)	− 4.5 ± 13.9 (n = 160)§	− 0.7 ± 15.3 (n = 159)	0.021
Week 52 (change from baseline)	− 6.0 ± 13.0 (n = 157)§	− 2.7 ± 17.4 (n = 157)	0.06
Week 78 (change from baseline)	− 5.6 ± 15.4 (n = 151)§	− 1.6 ± 18.3 (n = 152)	0.040
Week 104 (change from baseline)	− 5.3 ± 16.3 (n = 150)§	0.5 ± 18.0 (n = 148)	0.004
Diastolic blood pressure (mmHg)	77.7 ± 10.0 (n = 165)	79.1 ± 10.9 (n = 165)	0.22
Week 26 (change from baseline)	− 1.7 ± 8.9 (n = 160)*	0.1 ± 9.0 (n = 159)	0.08
Week 52 (change from baseline)	− 3.2 ± 9.3 (n = 157)§	− 0.8 ± 10.1 (n = 157)	0.028
Week 78 (change from baseline)	− 2.7 ± 9.5 (n = 151)§	− 1.1 ± 9.8 (n = 152)	0.15
Week 104 (change from baseline)	− 3.2 ± 10.1 (n = 150)§	− 1.0 ± 10.0 (n = 148)	0.05

Data are presented as mean ± SD or median (the 25th and 75th percentiles) values. Differences in parameters between groups at baseline were analyzed using Student’s t-test or Wilcoxon rank-sum test. Differences in parameters from baseline to week 52 and 104 within each group were analyzed by one-sample t-test or Wilcoxon signed-rank test. Differences in parameters from baseline to week 52 and 104 between groups were analyzed using Student’s t-test or Wilcoxon rank-sum test. *P < 0.05, ^#^P < 0.01, §P < 0.001

Tofogliflozin treatment, but not conventional treatment, significantly reduced the body mass index (BMI), waist circumference, and systolic and diastolic blood pressure relative to baseline. Improvements during observation period in BMI (− 1.0 ± 1.4 kg/m^2^ vs. − 0.2 ± 1.8 kg/m^2^, P < 0.001), waist circumference (− 1.2 ± 6.0 cm vs. 1.5 ± 4.3 cm, P < 0.001), and systolic blood pressure (− 5.3 ± 16.3 mmHg vs. 0.5 ± 18.0 mmHg, P = 0.004) were significantly larger in the tofogliflozin group than in the conventional group (Table 4). Tofogliflozin treatment, but not conventional treatment, significantly increased HDL-C levels relative to baseline within 26, 52, and 78 weeks, and the HDL-C levels at these examination points were significantly improved by tofogliflozin treatment than by conventional treatment. Although the total cholesterol levels from each examination week did not significantly vary, the reductions relative to baseline values tended to be larger in the conventional group (Table 4).

The eGFRs significantly decreased in both groups during the study. UAE significantly increased in the conventional group but tended to decrease in the tofogliflozin group, and the UAE level change was significantly greater in the conventional group than in the tofogliflozin group (Table 5).

**Table 5:** Effects of tofogliflozin on markers of renal function, inflammation, and cardio-vascular function

	Tofogliflozin group	Conventional group	P value
eGFR (mL/min/1.73 m^2^)	80.7 ± 20.8 (n = 168)	81.9 ± 24.0 (n = 169)	0.64
Week 26 (change from baseline)	− 2.9 ± 9.1 (n = 161)§	− 2.8 ± 9.4 (n = 162)§	0.89
Week 52 (change from baseline)	− 2.8 ± 9.5 (n = 159)§	− 2.3 ± 9.6 (n = 161)#	0.67
Week 78 (change from baseline)	− 3.2 ± 9.9 (n = 154)§	− 3.7 ± 10.4 (n = 157)§	0.65
Week 104 (change from baseline)	− 3.1 ± 12.1 (n = 155)#	− 3.8 ± 10.5 (n = 153)§	0.59
UAE at baseline (mg/g/cre)	13.0 [6.3, 37.0] (n = 158)	17.4 [5.8, 67.7] (n = 157)	0.24
Week 26 (change from baseline)	− 0.3 [− 8.9, 6.7] (n = 139)	− 0.4 [− 9.5, 4.5] (n = 134)	0.83
Week 52 (change from baseline)	− 1.7 [− 8.1, 3.9] (n = 146)*	0.1 [− 5.7, 9.6] (n = 146)	0.038
Week 78 (change from baseline)	− 0.8 [− 8.3, 8.0] (n = 131)	1.1 [− 6.4, 13.9] (n = 130)	0.06
Week 104 (change from baseline)	− 1.4 [− 9.9, 5.4] (n = 144)	1.8 [− 3.9, 20.1] (n = 140)*	0.006
hsCRP at baseline (ng/ml)	582.5 [287.5, 1430.0] (n = 168)	619.5 [295.0, 1450.0] (n = 170)	0.70
Week 52 (change from baseline)	− 24.5 [− 298.0, 190.0] (n = 154)	0.0 [− 260.0, 311.0] (n = 159)	0.41
Week 104 (change from baseline)	− 45.0 [− 412.5, 172.5] (n = 152)	18.5 [− 300.5, 305.0] (n = 152)	0.21
Adiponectin at baseline (μg/ml)	7.50 [5.30, 10.30] (n = 168)	7.35 [5.40, 10.30] (n = 170)	0.88
Week 52 (change from baseline)	0.65 [0.10, 1.30] (n = 154)§	0.40 [− 0.30, 1.10] (n = 159)§	0.019
Week 104 (change from baseline)	0.70 [− 0.10, 1.80] (n = 152)§	0.25 [− 0.30, 1.00] (n = 152)#	0.003
NT-proBNP at baseline (pg/ml)	34.0 [17.0, 68.0] (n = 168)	31.5 [17.0, 59.0] (n = 170)	0.96
Week 52 (change from baseline)	− 1.0 [− 14.0, 11.0] (n = 154)	− 2.0 [− 13.0, 9.0] (n = 159)	0.94
Week 104 (change from baseline)	1.0 [− 12.0, 17.0] (n = 152)	2.0 [− 8.5, 17.0] (n = 152)*	0.50

Data are presented as mean ± SD or median (the 25th and 75th percentiles) values. Differences in parameters between groups at baseline were analyzed using Student’s t-test or Wilcoxon rank-sum test. Differences in parameters from baseline to week 52 and 104 within each group were analyzed between groups using a one-sample t-test or Wilcoxon signed-rank test. Differences in parameter changes from baseline to week 52 and 104 were analyzed using Student’s t-test or Wilcoxon rank-sum test. *P < 0.05, ^#^P < 0.01, §P < 0.001

eGFR, estimated glomerular filtration rate; UAE, urinary albumin excretion; hs-CRP, high-sensitivity C-reactive protein; NT-proBNP, N-terminal pro–brain natriuretic peptide; baPWV, brachial-ankle pulse wave velocity; ABI, ankle brachial blood pressure index

Serum adiponectin levels significantly increased during the observation period in both groups, but its increase was more significant in the tofogliflozin group than in the conventional group. However, the hsCRP and NT-proBNP levels did not significantly change during the study (Table 5).

Remarkably, over the course of the study, the DPP-4 inhibitor use was significantly higher, and after 52 weeks, the metformin use was significantly higher in the conventional group than in the tofogliflozin group (Additional file 1: Table S2). Furthermore, antihypertensive drugs, especially angiotensin II receptor blockers (ARBs), were significantly more used, and the use of lipid-lowering agents tended to be higher in the conventional group than in the tofogliflozin group during the study (Additional file 1: Table S3).

### Adverse events

During the study, 168 patients, 76 in the tofogliflozin and 92 in the conventional group, developed adverse events (AEs), and 57 patients, 26 in the tofogliflozin and 31 in the conventional group, developed serious AEs. The total AE and serious AE incidences did not significantly vary between the treatment groups. Although a total 6 patients developed CV events, 3 patients in each group (hazard ratio, 0.96; 95% CI 0.21–5.03, P = 0.98), there were no CV-related deaths in either group during the follow-up period. A total of 34 hypoglycemic events, 17 in each group, were recorded (Table 6), but none of the affected patients experienced severe hypoglycemia. No significant variations in cancers, genital infections, urinary tract infections, or bone fractures were observed between the two groups, and no incidents of death, diabetic ketoacidosis, or leg amputation occurred.

**Table 6:** Summary of serious adverse events and adverse events

Parameters	Tofogliflozin treatment group (n = 169)	Conventional treatment group (n = 171)
Any adverse events	76 (45.0)	92 (53.8)
Severe adverse events	26 (15.4)	31 (18.1)
Death	0 (0)	0 (0)
Worsening of glycemic control	7 (4.1)	17 (9.9)
Hypoglycemia	17 (10.1)	17 (9.9)
Myocardial infarction	1 (0.6)	0 (0)
Other coronary artery diseases	3 (1.8)	1 (0.6)
Stroke	0 (0)	3 (1.8)
Subarachnoid hemorrhage	0 (0)	1 (0.6)
Peripheral artery disease	1 (0.6)	0 (0)
Heart failure	0 (0)	1 (0.6)
Arrhythmia	3 (1.8)	0 (0)
Blood pressure reductions	2 (1.2)	1 (0.6)
Venous thrombosis	0 (0)	1 (0.6)
Volume depletion	1 (0.6)	0 (0)
Gastric cancer	1 (0.6)	0 (0)
Hepatic cancer	2 (1.2)	0 (0)
Prostate cancer	1 (0.6)	1 (0.6)
Brest cancer	1 (0.6)	2 (1.2)
Colon cancer	1(1)	0 (0)
Malignant lymphoma	1 (0.6)	1 (0.6)
Anemia	1 (0.6)	3 (1.8)
Sleep apnea syndrome	0 (0)	1 (0.6)
Vertigo	2 (1.2)	2 (1.2)
ophthalmic diseases	3 (1.8)	7 (4.1)
Otolaryngology disease	7 (4.1)	4 (2.3)
Dental diseases	4 (2.4)	0 (0)
Influenza, common cold	22 (13.0)	23 (13.5)
Pneumonia	0 (0)	2 (1.2)
Other respiratory disease	2 (1.2)	2 (1.2)
Epigastric discomfort	3 (1.8)	1 (0.6)
Digestive tract disease	18 (10.7)	14 (8.2)
Liver dysfunction	1 (0.6)	1 (0.6)
Renal dysfunction	0 (0)	3 (1.8)
Urinary lithiasis	1 (0.6)	1 (0.6)
Dyslipidemia	0 (0)	3 (1.8)
Thyroid disease	3 (1.8)	1 (0.6)
Urinary tract infection	3 (1.8)	7 (4.1)
Genital infection	2 (1.2)	0 (0)
Genital pruritus	2 (1.2)	0 (0)
Dermatitis	2 (1.2)	1 (0.6)
Eruption	5 (3.0)	4 (2.3)
Muscle spasm	2 (1.2)	1 (0.6)
Bone fracture	3 (1.8)	3 (1.8)
Other orthopedic disease	4 (2.4)	12 (7.0)
Edema	0 (0)	2 (1.2)
General fatigue	1 (0.6)	1 (0.6)
Traumatic injury	0 (0)	3 (1.8)
Others	16 (9.5)	22 (12.9)

Data are presented as number (%) of patients

## Discussion

SGLT2 inhibitors, such as empagliflozin and canagliflozin, attenuated arteriosclerosis in animal models of the disease [31–33]. However, to date, there are no randomized controlled trials (RCT) monitoring the effect of SGLT2 inhibitors on the progression of arteriosclerotic lesions. Therefore, we conducted a PROBE study to investigate the preventive effects of a potent and selective SGLT2 inhibitor, tofogliflozin, on atherosclerosis progression in subjects with T2DM but no history of apparent CVD.

### Effects of tofogliflozin on atherosclerosis

To the best of our knowledge, the UTOPIA study is the first RCT evaluating the effects of SGLT2 inhibitor on carotid IMT. We found statistically significant IMT reduction in the tofogliflozin treatment group, and while the control group also showed a statistically significant IMT reduction, there were no significant differences in the progression in IMT between the two treatment groups.

IMT is a quantitative indicator of arteriosclerosis-related changes linked to CV risk factors that lead to the development of CVDs [34] and have been validated against pathological specimens and confirmed as strong predictors of CV events [35, 36]. Changes in the IMT values are used as an alternative index of CVD that can be measured repeatedly with low-cost, low-invasive techniques [16, 36–38]. IMT measurements have been applied as a surrogate outcome for evaluating the effects of various drugs, including statins and antidiabetic drugs, on arteriosclerosis in numerous clinical trials [17–24].

In this study, repeated IMT measurements were performed in a blinded manner and the analyses were performed at a core laboratory to avoid bias and measurement errors between institutions: all scans were electronically stored and sent to the core laboratory for reading by a single experienced reader unaware of the patients’ clinical characteristics in a random order using automated digital edge-detection software. The same procedure for analyzing carotid IMT was used in our previous studies [21, 22]. Thus, the reliability and reproducibility of IMT measurements were certified in the current study.

Two earlier, non-randomized studies evaluated the effects of SGLT2 inhibitors on IMT in T2DM patients [39, 40]. A 3-month prospective cohort study with 35 Italian T2DM patients by Irace et al. [39] showed that empagliflozin and incretin-based therapy reduced IMT. However, it did not evaluate potential differences in IMT reduction between groups, and was limited by small sample size and short treatment duration. The second trial was a single-arm intervention study in 134 Japanese T2DM patients who reported no statistically significant changes in IMT following 52 weeks of ipragliflozin treatment [40]. However, the report could not assess the effect of the SGLT2 inhibitors on IMT, since it was a single-arm study.

As the study drugs and participants’ backgrounds differ, the results of the above two studies cannot be directly compared with those of our UTOPIA study. However, our study is superior in terms of certain aspects including study design, sample size, and observation duration. Like Irace et al. [39], our study reports that IMT decreased after SGLT2 inhibitor treatment compared with pre-treatment. Moreover, all three studies report that SGLT2 inhibitor treatment has not been proven to significantly prevent IMT progression compared to with the conventional treatment.

The effects of SGLT2 inhibitors on endothelial function, which is an arteriosclerosis-related change that occurs on a shorter timescale than IMT hyperplasia [41], were evaluated in earlier reports. In the double-blind RCT EMBLEM, the effect of empagliflozin on the reactive hyperemia peripheral arterial tonometry index, an indicator of endothelial function, was estimated; however, the change was not significant [42]. Similarly, in another study, dapagliflozin did not significantly affect the flow-mediated dilation, another indicator of endothelial function [10]. These reports do not contradict our findings.

CVD onset events, such as myocardial infarction and stroke, occur later than IMT hyperplasia. Several cardiovascular outcome trials (CVOTs) with SGLT2 inhibitors have been published [6, 7, 43]. Remarkably, three studies reported substantial inhibition of heart failure, but there were no statistically significant inhibitory effects on events more closely related to atherosclerosis, such as fatal or non-fatal myocardial infarction [6, 7, 43]. Meta-analyses of CVOTs with new classes of antidiabetic drugs, including SGLT2 inhibitors, have produced similar findings [44], suggesting that possible mechanisms may involve hemodynamic effects induced by glycosuria and natriuresis rather than a direct antiatherothrombotic effect [5, 6, 8, 9].

In addition, it is possible that the effects of SGLT2 inhibitor treatment differ depending on vessels size. Both IMT and FMD are indices used to evaluate angiopathies in large vessels. Conversely, complex onset mechanisms underlie cardiovascular events, involving disorders of both large and small vessels. Coronary artery disease and stroke develop through complex onset mechanisms, in which disorders of both large and small vessels are involved but the presence of large-vessel lesions have greater significance. Previous studies have proven that SGLT2 inhibitors reduce mortality and hospitalization due to heart failure [6, 7, 43, 44]. Tofogliflozin has been reported to improve cardiac diastolic function [45]. The same study has also shown that the diastolic function improvement does not correlate with the improvement in FMD-based vascular endothelial function in large vessels and has inferred that tofogliflozin may improve diastolic function by improving local hemodynamics in the coronary artery. These findings suggest that the beneficial effects of SGLT2 inhibitors on small vessels may be greater rather than those on large vessels. This particular point warrants further investigation.

### Effects of tofogliflozin on glucose metabolism and other parameters

Within a 2-year observation period, relative to the control group parameters, tofogliflozin significantly reduced HbA1c, blood glucose, BMI, and abdominal circumference, which were accompanied by a significant HDL-C increase. These observations indicated that tofogliflozin is an effective drug for the management of CV risk factors in patients with T2DM.

In the tofogliflozin group, after 26 weeks, the HbA1c level was significantly decreased by an average of 0.4% from an initial level of 7.4 ± 0.7%, and even after 104 weeks, a significant average reduction of 0.3% was maintained. Since blood glucose was controlled in most subjects at the study start, the average reduction of 0.3–0.4% over 2 years appears to be clinically significant. Remarkably, the improvement in blood glucose control was accompanied by a significant reduction of body weight and abdominal circumference, but without an incident increase in hypoglycemic events. Since the beneficial effects of tofogliflozin on glycemic control has been reported to be more significant in subjects with larger visceral fat area at baseline, tofogliflozin would be more suitable for relatively obese subjects [46].

Tofogliflozin treatment was also associated with significant reduction of blood pressures and body weight, which were accompanied by a significant increase of red blood cell count, hemoglobin, and hematocrit. These findings were consistent with previous reports indicating the favorable hemodynamic effects of SGLT2 inhibitors [5–9]. SGLT2 inhibitor-induced plasma volume reduction and hemoconcentration leading to improvement of oxygen transport could contribute to the efficient myocardial energetics. Indeed, SGLT2 inhibitors including tofogliflozin significantly improve left ventricular diastolic function in patients with T2DM [45, 47]. It remains unclear whether there are differences in cardiac effects between agents belonging to this class of drugs, while a clinical comparison of tofogliflozin and empagliflozin based on a 48-week retrospective analysis showed that empagliflozin had a significantly stronger effect on the hematocrit than tofogliflozin [48].

### Safety

Although this study could not specifically evaluate the effects of tofogliflozin on CVD, the incidence of CV events was low in both treatment groups, and there was no significant difference between these groups. This incidence rate was similar to that reported in a recent large-scale RCT, J-DOIT3, that included Japanese T2DM patients [49].

In addition, all adverse events, hypoglycemia, genital infections, urinary tract infections, and bone fractures did not significantly vary between the two treatment groups. The fairly low incidence of adverse events related to genital infections and the absence of significant difference from the control group were consistent with the results of previous studies investigating tofogliflozin as a study drug in Japanese patients with T2DM [11, 12], although genital infections have been commonly known as an adverse event related to SGLT2 inhibitor treatment and thus should be administered with caution. Furthermore, there were no concerns documented in our study records about the safety of tofogliflozin.

These findings suggest that tofogliflozin is a safe and effective drug for managing T2DM in patients without apparent CVD.

### Limitations

Our study also has some limitations. First, although many clinical trials of antilipidemic and antidiabetic agents have used the carotid IMT as a surrogate clinical endpoint for cardiovascular events [17–24], there is no sufficient evidence whether the progression of carotid IMT reflects an increased risk of subsequent cardiovascular events. Some previous studies indicated that the progression of carotid IMT and carotid stenosis could be used as a surrogate endpoint of cardiovascular events [16, 36–38, 50, 51]. However, recent meta-analyses indicated that there was no significant association between the carotid IMT progression and the development of combined endpoints [52–55].

Second, this study was not a double-blind, placebo-controlled trial but rather a prospective, randomized study with open-label medications and blinded endpoint. Although the endpoint determination was blinded and conducted by expert committees, the medications were open label, which may induce unexpected bias. Furthermore, it is possible that the administration of additional antidiabetic, antilipidemic, and antihypertensive agents, which was more frequent in the control group at baseline and during the treatment periods (Additional file 1: Tables S2 and S3), may have affected the outcomes. These drugs presumably have direct anti-arteriosclerotic properties in addition to their effects on the reduction of blood glucose, blood pressure, and lipid levels. Specifically, biguanides [19, 20], DPP-4 inhibitors [21, 22], ARBs [23, 24], and statins [17, 18] exert strong inhibitory effects on the progression of IMT, and these effects are assumed to be independent of blood glucose, blood pressure, and lipid levels improvements. Thus, it is possible that the inhibition of IMT progression following tofogliflozin treatment might have been masked by the analogous effects of other drugs used in managing diabetes, as described above.

Third, we should consider that the relatively small number of subjects recruited for the study could be an important bias. Although the sample size had been considered as sufficient for detecting a difference in the IMT progression between the two treatment groups, it might have lacked the power to detect a smaller effect, which might be the reason why this study failed to observe a significant difference between the two treatment groups. The IMT progression rate observed for the conventional treatment group in our study was lower than the value expected based on a report by Yokoyama et al. [30] One possible explanation for this is that the mean HbA1c level (= 7.3%) in our control group was relatively better than that (= 7.9%) in the study by Yokoyama et al. since it comprised the results of meta-analysis of multiple studies conducted in early 2000s, which since then, the standard treatments of diabetes have improved. In addition, drugs anticipated to have vascular protective effects are currently being used more proactively. This may have resulted in a significant decrease in IMT progression rates in diabetic patients compared to rates during early 2000s. In addition, unlike Yokoyama et al., our participants were patients with no history of CVD; which it may be also related to a smaller IMT progression rate.

Fourth, there may have been measurement errors in IMT due to inter-sonographer differences, which were not evaluated in this study. However, IMT was measured by the same expert sonographer in each institution throughout all the visits based on the study protocol. In addition, we did not find significant heterogeneity in changes in IMT among institutions (data not shown).

Fifth, subjects in this study were Japanese patients with T2DM, a cohort with relatively low CV risks. Therefore, it would be premature to generalize our findings to other racial or ethnic groups. Furthermore, the average BMI of the study subjects was relatively high (27.0 ± 5.8 kg/m^2^ in the tofogliflozin and 27.0 ± 4.6 kg/m^2^ in the conventional group), since average BMI of Japanese patients with T2DM is approximately 25 kg/m^2^. Thus, there might have been some bias in the patient selection process during enrollment.

Finally, multiple statistical analyses were performed on these subjects, which would generate false-positive results derived from multiple testing. Thus, secondary endpoint results should be interpreted with caution.

## Conclusion

We found no differences in IMT change between the tofogliflozin and conventional treatment groups. However, tofogliflozin is a safe and effective treatment option for managing primary CVD risk factors, including high blood glucose levels in T2DM patients without apparent CVD.

## Supplementary information

**Additional file 1: Materials S1:** Safety evaluation. List of UTOPIA trial site investigators. **Table S1.** Effects of tofogliflozin on intima-media thickness were analyzed using covariance models. **Table S2.** Changes in concomitantly used anti-diabetic agents. **Table S3.** Changes in concomitantly used cardiovascular medications.

## Data Availability

The datasets generated and/or analyzed during our study will be available from the corresponding author on reasonable request.

## Abbreviations

AE: Adverse event
ANCOVA: Analysis of covariance
ARB: Angiotensin II receptor blocker
BMI: Body mass index
CV: Cardiovascular
CVD: Cardiovascular disease
CVOT: Cardiovascular outcome trial
DPP-4: Dipeptidyl peptidase-4
eGFR: Estimated glomerular filtration rate
IMT: Intima-media thickness
max-IMT-CCA: Maximum IMT of common carotid artery
mean-IMT-CCA: Mean IMT of common carotid artery
PROBE: Prospective, randomized, open label, blinded-endpoint
RCT: Randomized controlled trials
SGLT2: Sodium-glucose cotransporter 2
UAE: Urinary albumin excretion
UTOPIA: Using Tofogliflozin for Possible better Intervention against Atherosclerosis for type 2 diabetes patients
