# Supplementary material for: Tofogliflozin does not delay progression of carotid atherosclerosis in patients with type 2 diabetes: a prospective, randomized, open-label, parallel-group comparative study
Source: Cardiovasc Diabetol. 2020 Jul 9;19:110. doi: 10.1186/s12933-020-01079-4 (PMC7350187; doi:10.1186/s12933-020-01079-4)
Supplement: Supplementary file 1 — Additional file 1: Materials S1: Safety evaluation. List of UTOPIA trial site investigators. Table S1. Effects of tofogliflozin on intima-media thickness were analyzed using covariance models. Table S2. Changes in concomitantly used anti-diabetic agents. Table S3. Changes in concomitantly used cardiovascular medications. [file 12933_2020_1079_MOESM1_ESM.doc]

**ADDITIONAL MATERIALS**

**Safety evaluation**

All adverse events (AEs) were recorded during study. AEs were defined as any untoward medical occurrence in a clinical trial subject administered a medicinal product that were not necessarily related to this treatment. The details and incidence of all AEs were periodically ascertained. Based on the intention-to-treat the entire population, safety was checked by recording the AEs. When the investigators confirmed AEs, the severity grade, procedures, outcomes, and relationship to the study agent were assessed and reported to the trial organizer, the study secretariat, and the Data and Safety Monitoring Board (DSMB). The DSMB then deliberated on the incident and reported the decision to the chief investigator. Furthermore, serious AEs were reported to the principal investigator and the ethics committee. Both the investigator and the committee judged whether the diagnosis was appropriate or made a decision on whether the patient should be withdrawn from the trial. Cardiovascular events were diagnosed and fully assessed by members of the Cardiovascular Endpoint committee (which included two cardiologists and a neurologist).

**UTOPIA trial site investigators (listed in alphabetical order):**

*Hayashi Clinic:* I Hayashi

*Ikeda Municipal Hospital:* M Tsugawa

*Jiyugaoka Medical Clinic:* H Yokoyama

*Juntendo Tokyo Koto Geriatric Medical Center:* H Yoshii

*Juntendo University Graduate School of Medicine:* K Komiyama, T Mita, T Shimizu

*Kansai Rosai Hospital:* T Yamamoto

*Kanda Naika Clinic:* S Kawashima

*Kawasaki Hospital:* T Nakamura

*Kawasaki Medical School:* S Kamei, T Kinoshita, M Shimoda

*Kitasenri Maeda Clinic:* K Maeda

*Kosugi Medical Clinic:* K Kosugi

*Misaki Naika Clinic:* H Yoshii

*NakaKinen Clinic:* H Ishida, T Osonoi, M Saito, A Tamazawa

*Nissay Hospita:* S Sumitani

*Osaka General Medical Center:* N Fujiki, Y Fujita, S Shimizu, Y Umayahara

*National Hospital Organization Osaka National Hospital:* K Kato

*Osaka Police Hospital:* Y Irie, R Kataoka, T Yasuda

*Osaka Rosai Hospital:* Y Kiyohara, M Ohashi, K Ryomoto, Y Takahi

*Osaka University Graduate School of Medicine:* Y Fujishima, Y Fujita, A Fukuhara, K Fukui, Y Hosokawa, A Imagawa, H Iwahashi, K Mukai, N Katakami, T Katsura, D Kawamori, T Kimura, S Kobayashi, J Kozawa, F Kubo, N Maeda, T Matsuoka,K Miyashita, S Nakata, H Ninomiya, H Nishizawa, Y Okuno, M Otsuki, F Sakamoto, S Sasaki, I Sato, N Shimo, I Shimomura, M Takahara, T Takano, A Tokunaga, S Uno, M Yamaoka, S Yoneda

*Otoshi Medical Clinic:* K Ohtoshi

*Shiraiwa Medical Clinic:* T Shiraiwa

*University of Occupational and Environmental Health, Japan:* M Hajime, K Koikawa, F Kuno, A Kurozumi, K Matsushita, M Narisawa, K Tanaka, K Sugai, Y Okada, K Torimoto

**Table S1. Effects of tofogliflozin on intima-media thickness were analyzed using covariance models.**

|  | Tofogliflozin treatment group | Conventional treatment group | Treatment effect (tofogliflozin-conventional treatment) (mean change; 95%CI) |
| --- | --- | --- | --- |
| Common mean IMT (mean change; SE) | | |  |
| at week 52 | -0·080 (0·006) | -0·081 (0·005) | 0·001 (-0·012, 0·014), P=0·88 |
| at week 104 | -0·134 (0·007) | -0·140 (0·007) | 0·006 (-0·011, 0·023), P=0·50 |
| Right maximum IMT (mean change; SE) | | |  |
| at week 52 | -0·109 (0·012) | -0·122 (0·011) | 0·013 (-0·016, 0·041), P=0·38 |
| at week 104 | -0·157 (0·014) | -0·187 (0·013) | 0·029 (-0·003, 0·062), P=0·08 |
| Left maximum IMT at week 104 (mean change; SE) | | |  |
| at week 52 | -0·127 (0·018) | -0·107 (0·017) | -0·020 (-0·063, 0·023), P=0·36 |
| at week 104 | -0·202 (0·019) | -0·195 (0·018) | -0·007 (-0·053, 0·039), P=0·77 |

Differences in delta change in IMT from baseline between two groups were analyzed with analysis of covariance models that include treatment group, age, sex, use of insulin, baseline IMT, systolic blood pressure, and statin administration.

**Table S2. Changes in concomitantly used anti-diabetic agents.**

| **Parameters** | **Tofogliflozin treatment group** | **Conventional treatment group** | **P value** |
| --- | --- | --- | --- |
| Any concomitantly used anti-diabetic agents* |  |  |  |
| Baseline | 153 (90·5) | 152 (89·4) | 0·86 |
| Week 26 | 150 (89·8) | 155 (91·7) | 0·58 |
| Week 52 | 142 (88·8) | 149 (91·4) | 0·46 |
| Week 78 | 139 (88·5) | 149 (92·5) | 0·25 |
| Week 104 | 137 (87·8) | 144 (92·3) | 0·26 |
| Metformin |  |  |  |
| Baseline | 91 (53·8) | 100 (58·8) | 0·38 |
| Week 26 | 90 (53·9) | 105 (62·1) | 0·15 |
| Week 52 | 84 (52·5) | 105 (64·4) | 0·032 |
| Week 78 | 81 (51·6) | 104 (64·6) | 0·023 |
| Week 104 | 81 (51·9) | 104 (66·7) | 0·011 |
| Sulfonylurea |  |  |  |
| Baseline | 38 (22·5) | 43 (25·3) | 0·61 |
| Week 26 | 34 (20·4) | 43 (25·4) | 0·30 |
| Week 52 | 35 (21·9) | 46 (28·2) | 0·20 |
| Week 78 | 34 (21·7) | 41 (25·5) | 0·43 |
| Week 104 | 31 (19·9) | 41 (26·3) | 0·23 |
| Glinides |  |  |  |
| Baseline | 10 (5·9) | 10 (5·9) | 1·00 |
| Week 26 | 9 (5·4) | 11 (6·5) | 0·82 |
| Week 52 | 7 (4·4) | 9 (5·5) | 0·80 |
| Week 78 | 8 (5·1) | 10 (6·2) | 0·81 |
| Week 104 | 9 (5·8) | 9 (5·8) | 1·00 |
| Thiazolidinediones |  |  |  |
| Baseline | 18 (10·7) | 23 (13·5) | 0·51 |
| Week 26 | 17 (10·2) | 24 (14·2) | 0·32 |
| Week 52 | 18 (11·3) | 24 (14·7) | 0·41 |
| Week 78 | 17 (10·8) | 22 (13·7) | 0·50 |
| Week 104 | 17 (10·9) | 20 (12·8) | 0·73 |
| α-glucosidase inhibitors |  |  |  |
| Baseline | 24 (14·2) | 25 (14·7) | 1·00 |
| Week 26 | 25 (15·0) | 27 (16·0) | 0·88 |
| Week 52 | 24 (15·0) | 23 (14·1) | 0·88 |
| Week 78 | 25 (15·9) | 24 (14·9) | 0·88 |
| Week 104 | 24 (15·4) | 25 (16·0) | 1·00 |
| DPP-4 inhibitors |  |  |  |
| Baseline | 75 (44·4) | 95 (55·9) | 0·039 |
| 26 weeks | 72 (43·1) | 98 (58·0) | 0·009 |
| 52 weeks | 72 (45·0) | 93 (57·1) | 0·035 |
| 78 weeks | 71 (45·2) | 92 (57·1) | 0·043 |
| 104 weeks | 68 (43·6) | 89 (57·1) | 0·023 |
| GLP-1 R agonists |  |  |  |
| Baseline | 23 (13·6) | 12 (7·1) | 0·05 |
| Week 26 | 23 (13·8) | 14 (8·3) | 0·12 |
| Week 52 | 22 (13·8) | 16 (9·8) | 0·30 |
| Week 78 | 21 (13·4) | 14 (8·7) | 0·21 |
| Week 104 | 21 (13·5) | 13 (8·3) | 0·20 |
| Insulins |  |  |  |
| Baseline | 35 (20·7) | 37 (21·8) | 0·89 |
| Week 26 | 34 (20·4) | 37 (21·9) | 0·79 |
| Week 52 | 32 (20·0) | 39 (23·9) | 0·42 |
| Week 78 | 30 (19·1) | 38 (23·6) | 0·34 |
| Week 104 | 30 (19·2) | 36 (23·1) | 0·49 |

Data are number (%) of patients. The two treatment groups were compared by Fisher’s exact test. *; Administration of tofogliflozin in the tofogliflozin treatment group was not counted as concomitantly used anti-diabetic agents.

**Table S3. Changes in concomitantly used cardiovascular medications.**

|  | **Tofogliflozin treatment group** | **Conventional treatment group** | **P value** |
| --- | --- | --- | --- |
| Anti-hypertensive drugs |  |  |  |
| Any antihypertensive drugs |  |  |  |
| Baseline | 79 (46·7) | 95 (55·9) | 0·10 |
| Week 26 | 78 (46·7) | 98 (58·0) | 0·049 |
| Week 52 | 75 (46·9) | 97 (59·5) | 0·026 |
| Week 78 | 73 (46·5) | 96 (59·6) | 0·024 |
| Week 104 | 73 (46·8) | 92 (59·0) | 0·041 |
| Angiotensin-converting enzyme inhibitors |  |  |  |
| Baseline | 3 (1·8) | 5 (2·9) | 0·72 |
| Week 26 | 3 (1·8) | 5 (3·0) | 0·72 |
| Week 52 | 2 (1·3) | 5 (3·1) | 0·45 |
| Week 78 | 3 (1·9) | 5 (3·1) | 0·72 |
| Week 104 | 3 (1·9) | 5 (3·2) | 0·72 |
| Angiotensin II receptor blockers |  |  |  |
| Baseline | 63 (37·3) | 83 (48·8) | 0·037 |
| Week 26 | 62 (37·1) | 85 (50·3) | 0·016 |
| Week 52 | 61 (38·1) | 83 (50·9) | 0·025 |
| Week 78 | 58 (36·9) | 84 (52·2) | 0·007 |
| Week 104 | 56 (35·9) | 80 (51·3) | 0·009 |
| Calcium channel blockers |  |  |  |
| Baseline | 47 (27·8) | 54 (31·8) | 0·48 |
| Week 26 | 45 (26·9) | 55 (32·5) | 0·28 |
| Week 52 | 45 (28·1) | 57 (35·0) | 0·19 |
| Week 78 | 44 (28·0) | 58 (36·0) | 0·15 |
| Week 104 | 45 (28·8) | 57 (36·5) | 0·18 |
| Lipid-lowering agents |  |  |  |
| Any lipid-lowering agents |  |  |  |
| Baseline | 82 (48·5) | 99 (58·2) | 0·08 |
| Week 26 | 81 (48·5) | 99 (58·6) | 0·08 |
| Week 52 | 76 (47·5) | 99 (60·7) | 0·019 |
| Week 78 | 80 (51·0) | 99 (61·5) | 0·07 |
| Week 104 | 82 (52·6) | 98 (62·8) | 0·09 |
| Statins |  |  |  |
| Baseline | 73 (43·2) | 83 (48·8) | 0·33 |
| Week 26 | 72 (43·1) | 83 (49·1) | 0·28 |
| Week 52 | 67 (41·9) | 84 (51·5) | 0·09 |
| Week 78 | 70 (44·6) | 87 (54·0) | 0·09 |
| Week 104 | 73 (46·8) | 87 (55·8) | 0·14 |
| Antithrombotic agents |  |  |  |
| Any anti-thrombotic agents |  |  |  |
| Baseline | 17 (10·1) | 15 (8·8) | 0·71 |
| Week 26 | 17 (10·2) | 16 (9·5) | 0·86 |
| Week 52 | 16 (10·0) | 15 (9·2) | 0·85 |
| Week 78 | 16 (10·2) | 16 (9·9) | 1·00 |
| Week 104 | 16 (10·3) | 15 (9·6) | 1·00 |
| Antiplatelet drugs |  |  |  |
| Baseline | 15 (8·9) | 11 (6·5) | 0·42 |
| Week 26 | 15 (9·0) | 12 (7·1) | 0·55 |
| Week 52 | 14 (8·8) | 11 (6·7) | 0·54 |
| Week 78 | 14 (8·9) | 11 (6·8) | 0·54 |
| Week 104 | 14 (9·0) | 11 (7·1) | 0·68 |
| Anticoagulants |  |  |  |
| Baseline | 2 (1·2) | 4 (2·4) | 0·68 |
| Week 26 | 2 (1·2) | 4 (2·4) | 0·68 |
| Week 52 | 2 (1·3) | 4 (2·5) | 0·68 |
| Week 78 | 2 (1·3) | 5 (3·1) | 0·45 |
| Week 104 | 2 (1·3) | 4 (2·6) | 0·68 |

Data are presented as number (%) of patients or mean ± SD. The two treatment groups were compared by Fisher’s exact test.
